# Direct production of organic acids from starch by cell surface-engineered *Corynebacterium glutamicum* in anaerobic conditions

**DOI:** 10.1186/2191-0855-3-72

**Published:** 2013-12-17

**Authors:** Yota Tsuge, Toshihiro Tateno, Kengo Sasaki, Tomohisa Hasunuma, Tsutomu Tanaka, Akihiko Kondo

**Affiliations:** 1 Organization of Advanced Science and Technology, Kobe University, 1-1 Rokkodai, Nada, Kobe 657-8501, Japan; 2 Department of Chemical Science and Engineering, Graduate School of Engineering, Kobe University, 1-1 Rokkodai, Nada, Kobe 657-8501, Japan; 3 Biomass Engineering Program, RIKEN, 1-7-22 Suehiro-cho, Tsurumi-ku, Yokohama, Kanagawa 230-0045, Japan; 4 Department of Food Bioscience and Technology, College of Life Sciences and Biotechnology, Korea University, Seoul 136-713, Republic of Korea

**Keywords:** *Corynebacterium glutamicum*, Organic acid, α-amylase, Cell-surface engineering

## Abstract

We produced organic acids, including lactate and succinate, directly from soluble starch under anaerobic conditions using high cell-density cultures of *Corynebacterium glutamicum* displaying α-amylase (AmyA) from *Streptococcus bovis* 148 on the cell surface. Notably, reactions performed under anaerobic conditions at 35 and 40°C, which are higher than the optimal growth temperature of 30°C, showed 32% and 19%, respectively, higher productivity of the organic acids lactate, succinate, and acetate compared to that at 30°C. However, α-amylase was not stably anchored and released into the medium from the cell surface during reactions at these higher temperatures, as demonstrated by the 61% and 85% decreases in activity, respectively, from baseline, compared to the only 8% decrease at 30°C. The AmyA-displaying *C. glutamicum* cells retained their starch-degrading capacity during five 10 h reaction cycles at 30°C, producing 107.8 g/l of total organic acids, including 88.9 g/l lactate and 14.0 g/l succinate. The applicability of cell surface-engineering technology for the production of organic acids from biomass by high cell-density cultures of *C. glutamicum* under anaerobic conditions was demonstrated.

## Introduction

In the new era of green chemistry, the utilization of renewable resources, such as plant biomass, and environmentally friendly chemical products are eagerly desired. In particular, the organic acids lactic and succinic acids are recognized as ideal candidates for the building blocks of next-generation plastics. Both compounds were selected within the top 30 value-added chemicals manufactured from biomass by the U.S. Department of Energy (Werpy et al. 2004).

*Corynebacterium glutamicum* is a Gram-positive bacterium with a high G + C content and has been widely used in industry for the large-scale production of amino acids, including glutamate and lysine (Nakayama et al. 1961; Kinoshita 1985). Recently, *C. glutamicum* was shown to have increased metabolic flow through the glycolytic pathway and reductive branch of the tricarboxylic acid (TCA) cycle under oxygen-deprived conditions, resulting in the exclusive production of the organic acids lactate, succinate, and acetate (Inui et al. 2004). High cell-density cultivation promoted the consumption of oxygen and obviated the need for the removal of oxygen. Using this system, in which resting cell of *C. glutamicum* were cultured at high cell-density under anaerobic conditions, several organic acids, including lactate and succinate, were produced at high levels (Okino et al. 2005 Okino et al. 2008a Okino et al. 2008b Litsanov et al. 2012 Tsuge et al. 2013).

The cell-surface display of heterologous proteins has several potential biotechnological applications, including adsorption or degradation of environmental pollutants, recovery of rare metal ions, biosensors, and recombinant protein production (Kuroda and Ueda 2010, 2011). In addition to these applications, yeast and bacteria displaying fusion proteins composed of a cell surface-anchoring protein and a biomass-degrading protein have been effectively used for the fermentation of biomass resources, such as starch, xylan, cellobiose, and cellulose (Katahira et al. 2004; Matano et al. 2012; Murai et al. 1997; Narita et al. 2006; Yamada et al. 2013). The main advantage of cell surface-engineering technology is that cells displaying useful proteins can be reutilized multiple times, similar to biocatalysts (Matano et al. 2013; Yamakawa et al. 2012). We previously developed a cell surface-display system in *C. glutamicum* using the *Bacillus subtilis* PgsA and *C. glutamicum* PorC proteins as anchors for displaying α-amylase from *Streptococcus bovis* 148 and β–glucosidase from *Saccharophagus degradans* 2–40 for lysine production from starch and cellobiose, respectively, under growing conditions (Tateno et al. 2007a; Adachi et al. 2013). Although it was anticipated that the co-utilization of cell surface-engineering technology zand high cell-density systems would improve organic acid production from biomass, the efficacy of this combined approach in *C. glutamicum* has not been demonstrated.

In the present study, we demonstrated the application of cell surface-engineering technology for the production of organic acids by *C. glutamicum* cells cultured at high-density under anaerobic conditions in a minimal salts medium. Using this approach, organic acids, including lactate and succinate, were produced directly from starch by *C. glutamicum* displaying α-amylase on the cell surface. Moreover, we reutilized the *C. glutamicum* cells for the repeated production of organic acids to evaluate the stability and durability of the cell-surface displayed protein in *C. glutamicum*.

## Materials and methods

### Bacterial strains, media, growth conditions, and plasmids

All bacterial strains and plasmids used in this study are listed in Table 1. *Escherichia coli* NovaBlue was used for the construction of plasmids. *E. coli* SCS110 was used for the preparation of plasmid DNA for the transformation of *C. glutamicum. C. glutamicum* ATCC13032 was used as a host strain. *E. coli* strains were grown at 37°C in Luria-Bertani (LB) medium (Sambrook et al. 1989), and *C. glutamicum* and its recombinants were grown at 30°C in A+ medium (5 g yeast extract, 5 g casamino acids, 4 g urea, 14 g ammonium sulfate, 0.5 g KH_2_PO_4_, 0.5 g K_2_HPO_4_, 0.5 g MgSO_4_ · 7H_2_O, 20 mg FeSO_4_ · 7H_2_O, 20 mg Mn_2_SO_4_ · H_2_O, 0.2 mg biotin, and 0.2 mg thiamine per liter) supplemented with 2% glucose. When required, kanamycin was added at a concentration of 50 μg/ml for *E. coli* and at 25 μg/ml for *C. glutamicum*.

**Table 1:** Strains, plasmids, and primers used in this study

**Strain, plasmid or primer**	**Relevant genotype or description**	**Reference or source**
Strains		
*E. coli*		
NovaBlue	*endA1 hsdR17* (r_K12_^–^ m_K12_^+^) *supE44 thi-1 recA1 gyrA96 relA1 lac* F′[*proA*^*+*^*B*^*+*^*lacI*^*q*^*Z*Δ*M15*::Tn*10*] (Tet^r^)	Merck
SCS110	*dam, dcm supE44, hsdR17, thi leu, rpsL1, lacY, galK, galT, ara, tonA, thr, tsx,* Δ(*lac-proAB*)/*F’* [*traD36, proAB*^*+*^*, lacI*^q^*, lacZ*Δ M15]	Stratagene
*C. glutamicum*		
ATCC13032	Wild-type strain	ATCC
Plasmids		
pCC	Km^r^; *E. coli*-*C. glutamicum* shuttle vector containing *cspB* promoter	Tateno et al. 2007b
pCC-*pgsA*	Km^r^; pCC carrying *pgsA*	This study
pCC-*pgsA*-*amyA*	Km^r^; pCC-*pgsA* carrying *amyA* gene fused with 3’ terminal of *pgsA*	This study
Primers		
BamHI-pgsA_F	CGCGGATCCATGAAAAAAGAACTGAGCTTTCAT	This study
pgsA-SacI_R	ACGCGTCGACTTACTTGTCATCGTCATCCTTGTAGTCGAGCTC TTTAGATTTTAGTT TGTCACTATGATC	This study
SacI-amyA_F	CCCGAGCTCGATGAACAAGTGTCAATGAAAGAT	This study
amyA-XhoI_R	CCGCTCGAGTTATTTTAGCCCATCTTTATTATAGTTT	This study

*ATCC* American Type Culture Collection.

### Plasmid construction

All polymerase chain reactions (PCR) were performed using KOD-Plus-DNA polymerase (Toyobo Co., Ltd., Osaka, Japan). The *pgsA* gene was amplified by PCR using the plasmid pHLA (Narita et al. 2006) as template with primers BamHI-pgsA_F and pgsA-SacI_R (Table 1). The amplified fragment was digested with *Bam*HI and *Sac*I, and then ligated into *Bam*HI/*Sac*I digested plasmid pCC (Tateno et al. 2007b). The resulting plasmid was designated pCC-*pgsA*. The α-amylase gene (*amyA*) from *Streptococcus bovis* 148 was amplified by PCR using pCCS-*amyA* (Tateno et al. 2007b) as template and primers SacI-amyA_F and amyA-XhoI_R (Table 1). The amplified fragment was digested with *Sac*I and *Xho*I, and was then ligated into *Sac*I/*Xho*I digested pCC-*pgsA*. The resulting plasmid was designated pCC-*pgsA*-*amyA*.

### DNA manipulations

Transformation of *E. coli* was performed by the CaCl_2_ method. Transformation of *C. glutamicum* was carried out as described previously (Tateno et al. 2007a).

### Bioprocess conditions for organic acid production

For organic acid production, cells were harvested from 500 ml cultures in A+ medium by centrifugation (5000 × g, 4°C for 10 min) after 16 h cultivation at 30°C with starting at an optical density at 600 nm (OD_600_) of 0.05. The cell pellet was resuspended in 40 ml K10 medium, which consisted of 168 mg NH_4_H_2_PO_4_, 151 mg (NH_4_)_2_HPO_4_, 250 mg KCl, 400 mg MgSO_4_ · 7H_2_O, 16 mg FeSO_4_ · 7H_2_O, 16 mg MnSO_4_ · 7H_2_O, 0.16 mg biotin, and 0.16 mg thiamine (per liter), to give a final OD_600_ of 60. 40 ml of 24 g/l soluble starch, which was sterilized by autoclaving, was added to Bio Jr.8 fermentor (ABLE Biott, Japan), and the temperature was set at 30, 35, or 40°C. After the temperature had stabilized, 40 ml of the cell suspension was added to the bioreactor, resulting in a 80 ml culture with an OD_600_ of 30 and a final concentration of 12 g/l soluble starch. For repeated reactions, a 45 g/l soluble starch solution was used. After each reaction, cells were harvested by centrifugation (5000 × g, 4°C for 10 min) and then washed once with K10 medium. The cell pellet was resuspended in 40 ml of K10 medium, to which 40 ml of 90 g/l soluble starch solution was added, giving 80 ml cell suspensions containing 45 g/l soluble starch. Cell suspensions were agitated at 120 rpm without aeration. Anaerobic conditions were achieved by the rapid consumption of residual oxygen in the medium by cells. The pH of the medium was maintained at 7.0 throughout the reaction using a 5.0 N ammonia solution.

### Analytical procedures

OD_600_ was measured using a spectrophotometer (UVmini-1240; Shimadzu, Japan). Organic acids (lactate, succinate, and acetate) were quantified in centrifuged samples (15,000 rpm, 4°C, 10 min) using a high-performance liquid chromatograph (Shimadzu) equipped with a UV/VIS detector (SPD-20A) and a BioRad Aminex 87H column (Bio-Rad Laboratories, USA) operating at 50°C with a 5 mM H_2_SO_4_ mobile phase at a flow rate of 0.6 ml/min. The starch concentration was measured using an EnzyChrom Starch Assay Kit (Funakoshi) according to the manufacturer’s instructions.

### α-Amylase activity measurement

α-Amylase activity was measured with an α-amylase measurement kit (Kikkoman, Tokyo, Japan) as described previously (Tateno et al. 2007a). An OD_600_ of 1.0 corresponded to 0.39 mg dry weight cells ml^-1^.

## Results

### PgsA-anchored α-amylase sufficiently fermented soluble starch in high-cell density cultures of *C. glutamicum* cells

A *C. glutamicum* strain displaying PgsA-anchored α-amylase was previously shown to efficiently produce lysine from starch by direct simultaneous saccharification and fermentation under aerobic growing conditions (Tateno et al. 2007a). Here, we investigated if this system could be applied for the production of organic acids using high cell-density cultures of *C. glutamicum* cells under anaerobic conditions. We considered that the cell-surface display of α-amylase, as opposed to secretion, was ideal for the system because cells could be reutilized multiple times with high-cell density conditions. We fused the α-amylase-encoding *amyA* gene from *Streptococcus bovis* 148 with the C-terminal region of *pgsA* from *Bacillus subtilis* in pCC vector downstream of the *cspB* promoter, and then introduced the constructed vector (pCC-*pgsA*-*amyA*) into a wild-type *C. glutamicum* ATCC13032 strain. Wild-type *C. glutamicum* produces mainly lactate (1.79 mol/mol glucose) and smaller amounts of succinate (0.09 mol/mol glucose) and acetate (0.01 mol/mol glucose) under anaerobic conditions (Okino et al. 2005).

**Figure 1:**
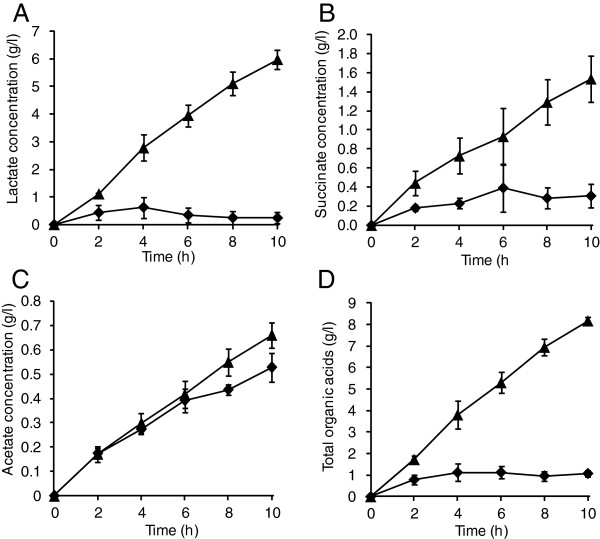
**Production of lactate (A), succinate (B), acetate (C), and total organic acids (D) from starch by *C. glutamicum *ATCC13032/pCC- *pgsA *-*amyA *(triangles) and ATCC13032/pCC-*pgsA *(diamonds).** Data points represent the averages calculated from three independent experiments. Standard deviations are indicated by bars or are within each symbol.

The recombinant strain expressing PgsA-anchored α-amylase on the cell surface was used for the bioproduction of organic acids directly from starch in a jar fermentor. The wild-type strain harboring pCC-*pgsA* was used as control. The pH and temperature of the jar fermentor were set at 7.0 and 30°C, respectively. The AmyA-displaying strain degraded starch and produced organic acids, including lactate, succinate, and acetate, at a relatively constant rate over the 10 h culture period (Figure 1). After 10 h of culture, the recombinant strain produced 6.0 ± 0.3 g/l lactate, 1.5 ± 0.2 g/l succinate, and 0.7 ± 0.1 g/l acetate (Table 2). The control strain also produced organic acids, albeit in smaller amounts, with concentrations of 0.2 ± 0.2 g/l lactate, 0.3 ± 0.1 g/l succinate, and 0.5 ± 0.1 g/l acetate detected after 10 h (Table 2). It is likely that these organic acids were generated by the control strain from the glucose formed during the heat sterilization of starch. Although the concentration of lactate in the control reactor reached as high as 0.6 ± 0.3 g/l after 4 h, the concentration decreased to 0.2 ± 0.2 g/l after 10 h indicating that the lactate was utilized as carbon source after the exhaustion of glucose due to the reversible catalytic activity of lactate dehydrogenase (LDH) (Figure 1A).

**Table 2:** Summary of organic acid production from starch at various temperatures

**Strain**	**Temperature (°C)**	**Starch consumed (g/l)**	**α-Amylase activity (U/g dry weight cell)**		**Concentration (g/l)**				**Yield**^**a**^
			**0 h**	**10 h**	**Lactate**	**Succinate**	**Acetate**	**Total organic acids**	
ATCC13032/pCC-*pgsA*	30	0.2 ± 0.2	0.1 ± 0.06	0.3 ± 0.05	0.2 ± 0.2	0.3 ± 0.1	0.5 ± 0.1	1.1 ± 0.1	–
ATCC13032/pCC-*pgsA*-*amyA*	30	9.2 ± 0.2	10.4 ± 1.0	9.5 ± 0.5	6.0 ± 0.3	1.5 ± 0.2	0.7 ± 0.1	8.2 ± 0.2	0.88 ± 0.01
ATCC13032/pCC-*pgsA*-*amyA*	35	12.8 ± 2.3	9.9 ± 1.0	3.9 ± 1.9	7.7 ± 0.5	2.1 ± 0.2	1.0 ± 0.1	10.8 ± 0.2	0.86 ± 0.13
ATCC13032/pCC-*pgsA*-*amyA*	40	12.0 ± 2.1	9.3 ± 0.1	1.4 ± 0.7	7.3 ± 1.2	1.5 ± 0.3	0.9 ± 0.1	9.7 ± 0.7	0.83 ± 0.13

^a^Yield is expressed as gram of total organic acids produced per gram of sugar consumed.

Each reaction was performed for 10 h.

Data represent the averages calculated from three independent experiments.

The AmyA-displaying strain mainly produced lactate (73% of total organic acids after 10 h), whereas the control strain predominantly generated acetate (45% of total organic acids after 10 h). Glucose remained below the limit of detection (data not shown) in the culture medium of the AmyA-displaying strain after 2 h, indicating that the glucose formed from starch by α-amylase was immediately transported into cells through the phosphoenolpyruvate:carbohydrate phosphotransferase system (Tanaka et al. 2008). The α-amylase activity in the AmyA-displaying strain was 10.4 ± 1.0 (U/g dry weight cell) at 0 h, but was only 0.1 ± 0.06 (U/g dry weight cell) in the control strain (Table 2). After 10 h of culture, the AmyA-displaying strain had retained its α-amylase activity, which was 9.5 ± 0.5 (U/g dry weight cell).

### Effects of temperature on starch degradation and organic acid production

The above-described bioprocess experiment was conducted at 30°C, which is the optimal growth temperature of *C. glutamicum*. However, because the optimum temperature for *S. bovis* AmyA activity is 50°C *in vitro* (Satoh et al. 1993), we also examined the effect of high temperature (35 and 40°C) on organic acid production by the recombinant strain from starch. In addition, performing fermentations at high temperature would reduce production costs associated with cooling the reactor, as the metabolic activities of microorganisms typically generate heat (Abdel-Banat et al. 2010).

High-density cell suspensions, which had an OD_600_ of 30 and a total volume of 80 ml, were cultured at 35 and 40°C under anaerobic conditions. Under these conditions, starch was efficiently degraded by the AmyA-displaying strain and organic acids were produced. Starch consumption was highest (12.8 g/l) at 35°C after 10 h, representing a 39% increase compared to that consumed at 30°C (Table 2). Interestingly, a similar amount of starch was degraded at 40°C, even though the cells barely grew at this temperature. Glucose was not detected at either of the higher temperatures, indicating it was immediately imported into cells and consumed after the degradation of starch. The total amount of produced organic acids was 32% and 18% higher at 35 and 40°C, respectively, than at 30°C (Table 2). In addition, the ratio of produced organic acids did not markedly change depending on the reaction temperature. Notably, however, α-amylase activity on the cell surface decreased by 61% after 10 h at 35°C, whereas a decrease of only 8% was observed at 30°C (Table 2). AmyA-displaying cells cultured at 40°C exhibited an even higher decrease (85%) of α-amylase activity. These results indicated that AmyA proteins were stably anchored on the cell surface at 30°C, whereas most anchored AmyA proteins were released into the medium at 35 and 40°C. The results also showed that although AmyA proteins were not anchored on the cell surface at 35 or 40°C, the metabolic activity of *C. glutamicum* cells was maintained.

### Repeated production of organic acids from starch

Cell-surface engineering is an ideal technology for the repeated use of cells, but only if the displayed protein maintained activity after several culture cycles and associated centrifugation steps, and medium exchange. Here, the ability of cell-surface engineered *C. glutamicum* cells to produce organic acids in repeated culture cycles was therefore investigated.

Cell suspensions of ATCC13032/pCC-*pgsA*-*amyA* strain with an OD_600_ of 159 were used for the production of organic acids from 50 g/l soluble starch as the sole carbon source in the first 10 h cycle. The temperature was set at 30°C for all cycles based on the stable α-amylase activity observed in the initial experiments. After the completion of the first cycle, 33.2 g/l organic acids (27.6 g/l lactate, 3.9 g/l succinate, and 1.3 g/l acetate) were produced from 39.5 g/l starch (Table 3). The reaction mixture was then centrifuged and the collected cells were washed once with K10 medium. Cells were then resuspended in a total of 80 ml K10 medium supplemented with 45 g/l soluble starch. The repeated bioproduction of organic acids by the recombinant strain was performed for an additional four cycles. The initial OD_600_ of the cultures gradually decreased with increasing cycle number due to cell death and loss during the washing and centrifugation steps. However, the ratio of starch consumption per total cell mass between the first and last cycles was 1 to 0.77, showing that major of the displayed α-amylase retained activity after 50 h. During the total 50 h culture period, with consisted of five cycles in the bioreactor, 128.5 g/l starch was consumed and 107.8 g/l organic acids were produced, including 88.9 g/l lactate and 14.0 g/l succinate with the yield of 0.84 g/g (Table 3).

**Table 3:** **Production of organic acids from starch by *C. glutamicum *** **cells in repeated cycles**

**Cycle**	**Start OD**_**600**_	**Consumed starch (g/l)**	**α-Amylase activity (U/g dry weight cell)**		**Concentration (g/l)**				**Yield**^**a**^
			**0 h**	**10 h**	**Lactate**	**Succinate**	**Acetate**	**Total organic acids**	
1	159	39.5	15.4	9.4	27.6	3.9	1.3	32.8	0.83
2	122	27.5	9.5	7.2	19.5	2.8	0.8	23.2	0.84
3	103	23.0	7.0	5.4	15.6	2.4	0.9	18.9	0.82
4	99	21.2	7.8	5.4	14.4	2.7	1.1	18.3	0.86
5	91	17.3	6.8	5.0	11.8	2.2	0.8	14.8	0.86

^a^Yield is expressed as gram of total organic acids produced per gram of sugar consumed.

Each cycle was performed for 10 h at 30°C.

## Discussion

In the present study, we demonstrated the applicability of cell surface-engineering technology in *C. glutamicum* for production of the organic acids lactate and succinate from high-density cultures under anaerobic conditions. Although the *Streptococcus bovis* 148 AmyA protein used in this study has optimal *in-vitro* activity at 50°C, we demonstrated that the *in vivo* production of organic acids from starch could be achieved at 35 or 40°C, which is markedly higher than the optimal growth temperature of 30°C for *C. glutamicum*. At these temperatures, the consumption of starch and production of organic acids were accelerated with increasing reaction temperature. In a previous study examining lysine production from starch by *C. glutamicum* cells expressing PgsA-anchored α-amylase, cultivation at 40°C resulted in severe growth defects that were accompanied by dramatic decreases in lysine production and starch consumption compared to those observed at 30, 34, and 37°C (Tateno et al. 2007a). This difference was apparently because lysine production was performed under growing conditions. In contrast, the resting-cell reactions performed here with high cell-density cultures of *C. glutamicum* maintained their metabolic capacity despite the apparent lack of cell growth at higher temperature, and showed even higher starch consumption and organic acid production. Therefore, the optimal temperatures for cell growth and the central metabolic pathway that converts glucose to lactic acid, succinic acid, and acetic acid are likely different. However, because the ratio of produced organic acids did not alter dramatically at different temperatures, the optimal temperature for enzymes in the central metabolic pathway of *C. glutamicum* is likely to be similar. In resting cells of *Brevundimonas diminuta*, the production of ascorbic acid-2-phosphate was enhanced at 40°C compared to the optimal growth temperature of 30°C (Shin et al. 2007). Production of chemicals and fuels at higher temperature is advantageous for industrial processes, because it would reduce the risk of contamination during fermentation. More directly, higher temperatures would reduce the costs for cooling of the reactor. For example, a 5°C increase in the fermentation temperature would reportedly save approximately $30,000 per year for a 30 m^3^ scale plant (Abdel-Banat et al. 2010).

The AmyA protein fused with the C-terminus of cell-surface protein PgsA remained stable anchoring to the cell surface after several reaction cycles at 30°C, which included cell washing and medium exchange events. This finding contrasted that of the previous study on lysine production showing that cell-surface expressed AmyA protein was released into the medium at 30°C. There are two possible explanations for this difference. First, a certain proportion of fusion α-amylase proteins may be firmly anchored on the cell surface, whereas other fusion proteins failed to anchor after being transported to the cell surface. The second possibility is that stable anchoring of the fusion protein might be dependent on cell division, as we observed that organic acid production occurred in the absence of apparent cell growth. In either case, screen of more stable anchoring proteins would be desired to display α-amylase, or other protein of interest, on the cell surface of *C. glutamicum* cells. More stable anchoring proteins could lead to enhance process productivity at high temperature with repeated use of *C. glutamicum* cells, that could not be obtained by protein secretion.

In conclusion, we demonstrated that cell surface-engineering technology was advantageous for the repeated use of *C. glutamicum* cells cultured at high cell-density for organic acid production. This approach could be applied for displaying other biomass-degrading enzymes for the direct production of organic acids from renewable biomass.

## Competing interests

The authors declare that they have no competing interests

## Authors’ contributions

YT performed bioprocess reaction, enzyme assay and wrote the manuscript. TTateno designed and constructed plasmids and strains. KS performed HPLC analysis. TH and TTanaka critically revised the manuscript. AK supervised on the manuscript. All authors read and approved the final manuscript.
